# CD27- IgD- B cell memory subset associates with inflammation and frailty in elderly individuals but only in males

**DOI:** 10.1186/s12979-019-0159-6

**Published:** 2019-08-13

**Authors:** Tapio Nevalainen, Arttu Autio, Laura Kummola, Tanja Salomaa, Ilkka Junttila, Marja Jylhä, Mikko Hurme

**Affiliations:** 1 Faculty of Medicine and Health Technology, Tampere University, FI-33014 Tampere, Finland; 2 Faculty of Social Sciences, Tampere University, Tampere, Finland; 3 Gerontology Research Center, Tampere University, Tampere, Finland; 4 Science Centre, Pirkanmaa Hospital District, Tampere, Finland; 5 Department of Clinical Microbiology, Fimlab Laboratories, Tampere, Finland

**Keywords:** Immunosenescence, B cell, Aging, Frailty

## Abstract

**Background:**

Immunosenescence, i.e. the aging-associated decline of the capacity of the immune system, is characterized by several distinct changes in the number and functions of the immune cells. In the case of B cells, the total number of CD19+ B cells is lower in the blood of elderly individuals than in the younger ones. CD19+ B cell population contains several subsets, which are commonly characterized by the presence of CD27 and IgD molecules, i.e. naïve B cells (CD27- IgD+), IgM memory (CD27+ IgD+), switched memory (CD27+ IgD-) and late memory (CD27- IgD-). This late memory, double negative, population represents cells which are nondividing, but are still able to produce inflammatory mediators and in this way maybe contributing to the aging-associated inflammation, inflammaging. Here we have focused on the role of these B cell subsets in elderly individuals, nonagenarians, in the regulation of inflammation and inflammation-associated decline of bodily functions. As the biological aging process demonstrates gender-specific characteristics, the analyses were performed separately in males and female.

**Results:**

A subcohort of The Vitality 90+ study (67 nonagenarians, 22/45 males/females and 40 young controls, 13/27 males/females) was used. Flow cytometric analysis indicated that the total percentage of the CD19+ cells was ca. 50% lower in the nonagenarians than in the controls in both genders. The proportions of these four B cell subsets within the CD19+ populations were very similar in young and old individuals. Thus, it seems that the aging-associated decline of the total CD19+ cells affects similarly all these B cell subsets. To analyze the role of these subsets in the regulation of inflammation, the correlation with IL-6 levels was calculated. A significant correlation was observed only with the percentage of CD27- IgD- cells and only in males. As inflammation is associated with aging-associated functional performance and frailty, the correlations with the Barthel index and frailty score was analyzed. Again, only the CD27- IgD- population demonstrated a strong male-specific correlation.

**Conclusions:**

These data show that the CD27- IgD- B cell subset demonstrates a strong pro-inflammatory effect in nonagenarians, which significantly associates with the decline of the bodily functions. However, this phenomenon is only observed in males.

## Background

The impact of aging on the function of the immune system is commonly called immunosenescence. Both the changes at the level of individual cells of the immune system as well as the clinical diseases associated with its presence are relatively well characterized (for recent reviews, see [1, 2]). The immune system contains several functional entities such as innate/non-adaptive immune response, inflammation, and adaptive (B and T cell mediated) immune response and all of these are clearly affected by aging.

One distinct component of immunosenescence is the increased rate of inflammation, often called inflammaging (reviewed in [3]). It is a chronic, sterile, low grade inflammation that is manifested by an increase of several markers of inflammation in the blood of elderly individuals. It is currently recognized as the main driving force of the aging-associated phenotypic changes and a significant risk factor for the aging-associated diseases (reviewed in [4]). Although these adverse effects of inflammaging are now better characterized, the factors inducing inflammaging or modulating its activity are still, at least partly, unknown. These factors could be categorized as follows: i) infectious agents not eliminated by the aging immune system ii) cellular debris released from an ongoing degenerative disease (in some cases this would indicate reverse causality, i.e. inflammation is a consequence rather than cause of this disease process) iii) environmental and life-style factors iv) aging-associated increase or activity of a given cell type having a pro-inflammatory activity.

There are four relatively well-characterized CD19+ cell subtypes in the human peripheral blood i.e. naïve (CD27- IgD+), IgM memory (CD27+ IgD+), switched memory (CD27+ IgD-) and late memory (CD27- IgD-) [5]. Although there are obvious inter-individual differences and variation in the analysis methods it seems that the total percentage of CD19+ cells is decreased in aged individuals and consequently, also the number of the cells in these subsets is lower [5]. It is very likely that the activity of the CD27- IgD- subset contributes to inflammaging as it has the characteristics of the senescence-associated secretory phenotype (SASP) (nondividing, metabolically active and secreting proinflammatory cytokines) (reviewed in [6]). SASP phenotype in the cells of the immune system is probably a consequence of the general cellular senescence, which is known to be involved in the pathogenesis of many common diseases (e.g. diabetes, hypertension, tumorigenesis) (reviewed in [7]). In the case of immune/inflammatory diseases increased SASP activity has been observed in several infections and autoimmune diseases (reviewed in [8]).

In this study, we analyzed the significance of the CD27- IgD- B cells on the development of the serious aging-associated condition, i.e. frailty.

## Methods

### Study population

The study population consisted of 67 nonagenarians (22 males, 45 females) and 40 young controls (age 19–29 years, 13 males, 27 females) who were participants in the Vitality 90+ study, an ongoing population-based study involving individuals aged 90 years or older and living in the city of Tampere, Finland. The recruitment and characterization of the participants in this cohort have been characterized [9, 10]. The study protocol was approved by the ethics committee of the city of Tampere (1592/403/1996) and the study was conducted according to the principles expressed in the declaration of Helsinki. Collection of the blood samples and purification of peripheral blood mononuclear cells (PBMC) have recently been published in detail [11].

### Flow cytometry

After thawing, samples were washed with 1% fetal bovine serum (FBS) in phosphate-buffered saline (PBS) and incubated with Human BD Fc Block™ (BD, Franklin Lakes, NJ, US) for 10 min at room temperature. Cells were then surface stained with a mixture of the following mouse anti-human antibodies (all eBioscience™ from Thermo Fisher Scientific, Waltham, MA, US): FITC-IgD (clone IA6–2), PE-CD43(clone eBio84-3C1), PerCP-Cy5.5-CD19 (clone HIB19), PE-Cy7-CD27 (clone C323), APC-eFluor780-CD3 (clone UCHT1), APC-eFluor-CD14 (clone 61D3), APC-eFluor780-CD56 (clone CMSSB) and APC-CD20 (clone 2H7). After 20 min at 4 °C, the cells were washed twice with 1% FBS in PBS and samples were analyzed with FACSCanto II (BD). Further analysis was performed with FlowJo software (Ashland, OR, US). Gating was based on fluorescence minus one controls with slight adjustments according to individual variation. Debris and clearly apoptotic cells were excluded by FSC/SSC gating, but due to limitations in flow cytometer detector availability, staining for viability and CD45 was not performed. The PE-CD43 and APC-CD20 antibodies were included in the original antibody mixture for purposes outside the scope of this report.

### Assessment of functional performance and frailty

Physical performance in everyday life was assessed using the 10-item Barthel index [12]. It includes the activities required for daily living (such as mobility, dressing, feeding, toilet use, bowel and bladder control). Each task was scored individually (0–10) and the total score of 100 indicates maximum independence and the score 0 reflects total dependence. The frailty index was calculated as described in detail by Fried et al. [13]. The criteria included 1) mini-mental state examination (MMSE) score 2) weight loss 3) self-reported fatigue 4) hand grip strength 5) mobility. The sum of these criteria gives the final index (min 0, max 5).

### IL-6 quantification

The plasma interleukin 6 (IL-6) concentration was measured using the Pelikine human IL-6 ELISA kit (Sanguin, Amsterdam, The Netherlands).

### Statistical analysis

The differences in proportions of CD19+ cells, in proportions of B cell subsets, and plasma IL-6 levels between nonagenarian group and young group were analyzed using Mann-Whitney U test. Spearman’s Rank-Order correlation was used to test the association of B cell subsets with plasma IL-6 levels, Barthel index and Frailty index. All statistical analyses were performed using IBM SPSS Statistics version 25.

## Results

**Fig. 1:**
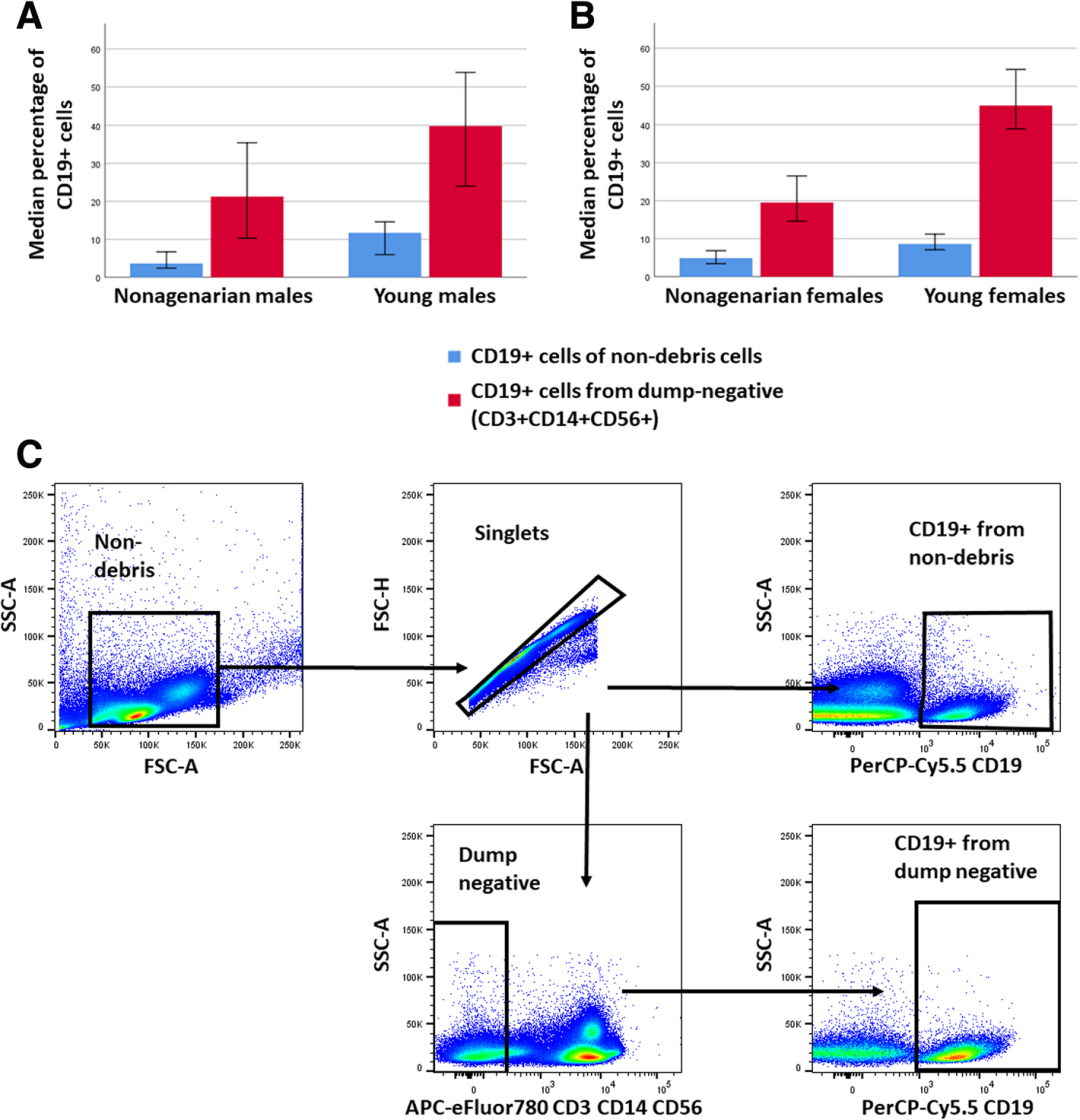
CD19+ B cell proportions and gating strategy. Comparison of the median CD19+ B cell proportions in the PBMCs of nonagenarian individuals and young individuals was analyzed with gating directly to CD19 from non-debris gate (blue) and with first gating out the CD3+, CD14+ and CD56+ (red). Error bars represent 95% confidence interval. **a** Median B cell proportions in nonagenarian males and young males. Significance for the age difference was *p* = 0.02 for the non-debris CD19+ cells and *p* = 0.011 for the dump-negative CD19+ cells. **b** Median B cell proportions in nonagenarian females and young females. Significance for the age difference was *p* < 0.001 for the non-debris CD19+ cells and p < 0.001 for the dump-negative CD19 + cells. **c** Gating strategy for CD19+ B cells. The percentage of CD19+ cells was determined after exclusion of debris (non-debris gate) and cell doublets (singlets gate), or after additionally gating out cells that express T cell, NK cell and monocyte lineage markers CD3, CD56 and CD14, respectively (“dump channel”, dump negative gate)

As reported in previous studies, the percentage of CD19+ B cells was clearly reduced in the elderly individuals as compared to the young controls. In the flow-cytometric analysis this was observed both by gating directly the CD19+ cells and after first gating out the CD3+, CD14+ and CD56+ cells. This reduction was slightly stronger in females (*p* < 0.001, using both gating strategies) than in males (*p* = 0.002 and *p* = 0.011 with direct gating and with gating out the dump channel (CD3, CD56 and CD14), respectively) (Fig. 1.).

**Fig. 2:**
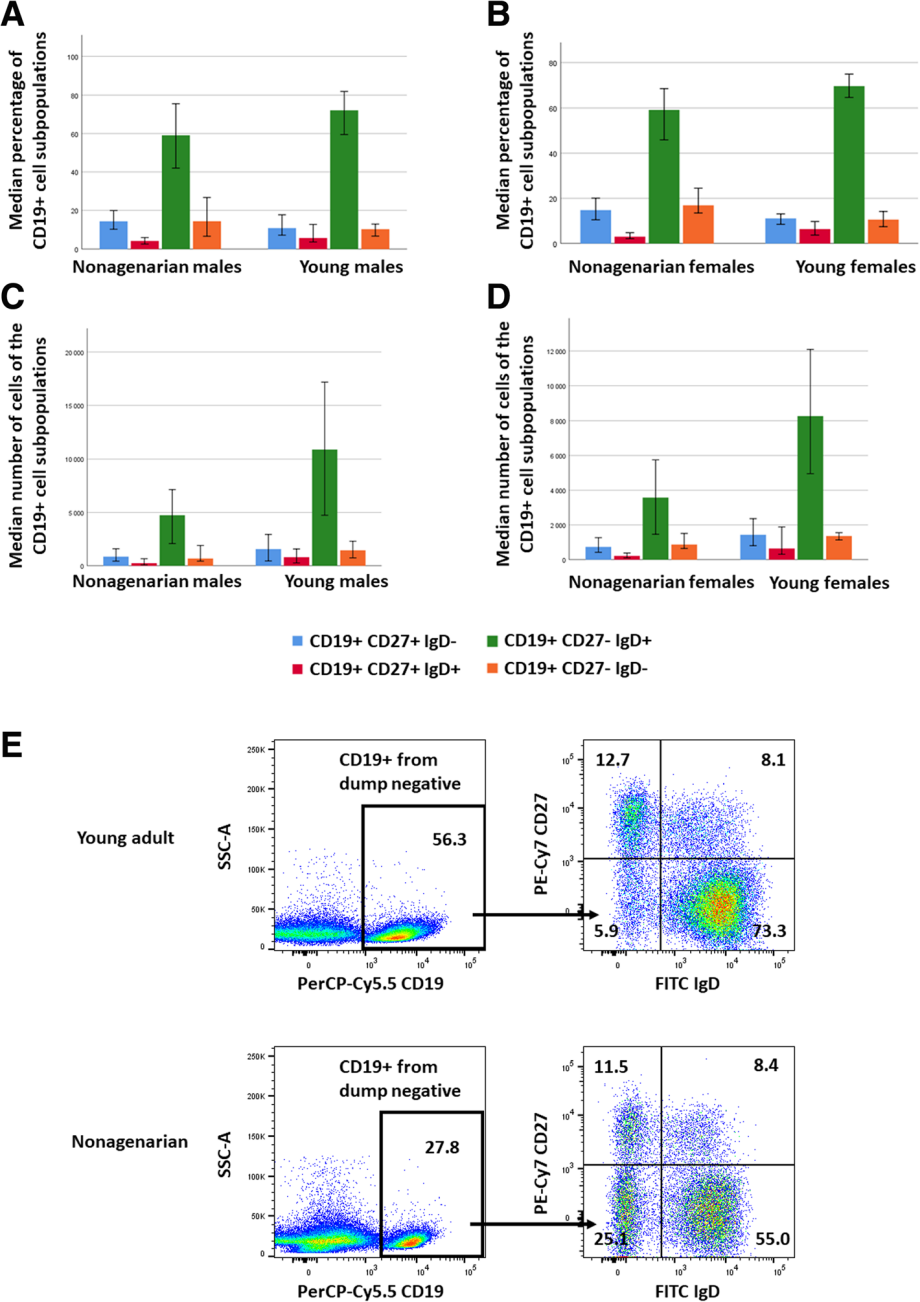
Genderwise comparison of the distributions of the CD19+ B cell subpopulation proportions and the CD19+ subpopulation gating strategy. **a** Median B cell subpopulation proportions in nonagenarian males and young males. *P*-values of the significances for the aging-associated differences were 0.113, 0.159, 0.057 and 0.139 for CD27+ IgD-, CD27+ IgD+, CD27 -IgD+ and CD27- IgD-, respectively. **b** Median B cell subpopulation proportions in nonagenarian females and young females. P-values of the significances for the aging-associated differences were 0.083, 0.002, 0.022 and 0.009 for CD27+ IgD-, CD27+ IgD+, CD27 -IgD+ and CD27- IgD-, respectively. Error bars represent 95% confidence interval. **c** Median B cell subpopulation cell numbers in nonagenarian males and young males. **d** Median B cell subpopulation cell numbers in nonagenarian females and young females. **e** Gating strategy of CD19+ B cell subpopulations. Representative samples from a young adult and a nonagenarian donor are shown. Numbers indicate population frequencies

The proportions of the CD27- IgD-, CD27- IgD+, CD27+ IgD+ and CD27+ IgD- subpopulations are shown in Fig. 2. It shows that the naïve B cells (CD27- IgD+) form the majority in both young and old individuals and in both genders.

In the case of the three memory cell populations the general pattern of the proportions (Fig. 2.) indicates that age and gender associated differences are minor. However, in females the higher proportion of CD27-IgD- cells (*p* = 0.009) and lower proportions of CD27+ IgD+ cells (*p* = 0.002) and CD27- IgD+ cells (*p* = 0.022) in nonagenarians as compared to controls were statistically significant. There was no significant aging associated difference in the CD27+ IgD- subpopulation. In the case of males, all age associated differences in the CD19+ subpopulation proportions were non-significant (*p* = 0.113, *p* = 0.159, *p* = 0.057 and *p* = 0.139 for CD27+ IgD-, CD27+ IgD+, CD27 -IgD+ and CD27- IgD-, respectively).

Taken together, the data this far shows that aging does not have a profound effect on the relative proportions of these B cell subsets; as the total number of B cells is clearly reduced (ca. 50%, see Fig. 1.) it can be concluded that also the total cell number of the four subsets are reduced in the aged individuals. To confirm that age associated reduction in CD19+ cells affects all CD19+ subpopulations, cell numbers, from which the subpopulation proportions were calculated, were also examined. The distributions of the cell numbers of the CD19+ subpopulations are shown in Fig. 1 (panels C and D). These distributions are clearly similar in shape over gender and age. Only notable difference seems to be the overall magnitude of the cell numbers over the age, that is explained by the difference in the number of CD19+ cells between nonagenarians and young individuals. This concludes that the age associated decrease in the CD19+ cells affects all the studied CD19+ subpopulations.

**Fig. 3:**
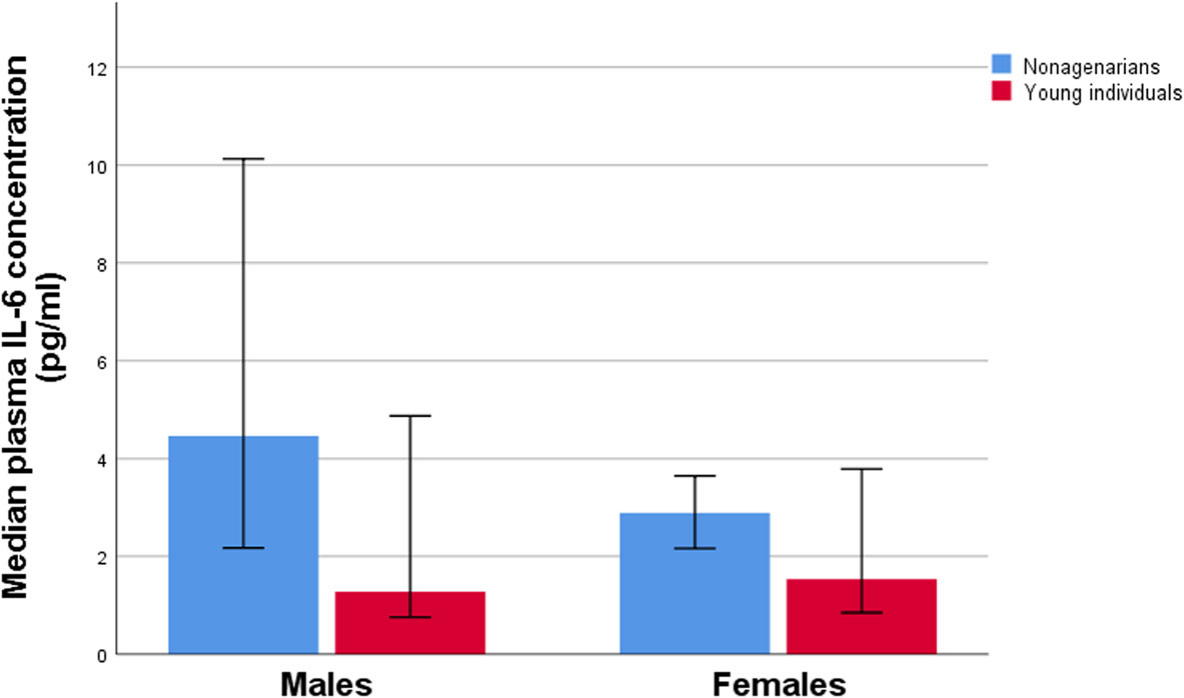
Genderwise comparison of median plasma Interleukin-6 concentrations in nonagenarian individuals and young individuals. IL-6 levels were significantly increased in nonagenarian males as compared to young males (*p* = 0.007), and in nonagenarian females as compared to young females (*p* = 0.010). Error bars represent 95% confidence interval

To examine the functional role of these B cell subsets, their associations with the serum levels of IL-6, i.e. the prototype proinflammatory cytokine, were analyzed. Figure 3 demonstrates that IL-6 levels were increased in the elderly individuals, both in males (*p* = 0.007) and females (*p* = 0.010). The data shown in Table 1 demonstrate that these levels correlated only with the proportion of the CD27- IgD- subset but only in males; thus, in males, the higher proportion of the cells were CD27- IgD-, the higher the plasma IL-6 level was.

**Table 1:** Spearman correlation of the plasma IL-6 levels (pg/ml) with the proportions of the B cell subsets in nonagenarians (22 males, 39 females)

B cell subset	gender	correlation coefficient	p-value
CD27- IgD-	male	**0.666**	**< 0.001**
	female	0.183	0.265
CD27- IgD+	male	−0.253	0.256
	female	−0.150	0.362
CD27+ IgD+	male	−0.217	0.331
	female	0.004	0.982
CD27+ IgD-	male	−0.300	0.175
	female	0.015	0.926

Correlation coefficient and significance of statistically significant associations are indicated in boldface

Aging is associated with a decline of the physical capacity in everyday life as well as increased vulnerability to endogenous and exogenous stressors. These changes are commonly quantitated using the Barthel and frailty indices, respectively. As inflammation is known to be associated with these parameters, we next analyzed the correlations of the B cell subsets with these indices. Table 2 shows that both of these indices correlate significantly only with the percentage of the CD27- IgD- population but only in males. Thus, this cell population seems to be involved in the mechanisms leading to the decline of functional capacity (negative correlation with the Barthel index) and increasing frailty.

**Table 2:** Spearman correlation of the Barthel and frailty indices with the proportions of the B cell subsets in nonagenarians

B cell subset	gender	r (Barthel index)	p-value (Barthel index)	r (Frailty index)	p-value (Frailty index)
CD27- IgD-	male	**−0.601**	**0.003**	**0.616**	**0.002**
	female	−0.274	0.091	0.157	0.34
CD27- IgD+	male	0.180	0.423	−0.387	0.076
	female	0.332	0.039	−0.181	0.27
CD27+ IgD+	male	0.339	0.123	−0.148	0.512
	female	−0.173	0.293	−0.113	0.492
CD27+ IgD-	male	0.358	0.102	−0.036	0.874
	female	−0.256	0.116	−0.003	0.983

Correlation coefficient and significance of statistically significant associations are indicated in boldface

**Fig. 4:**
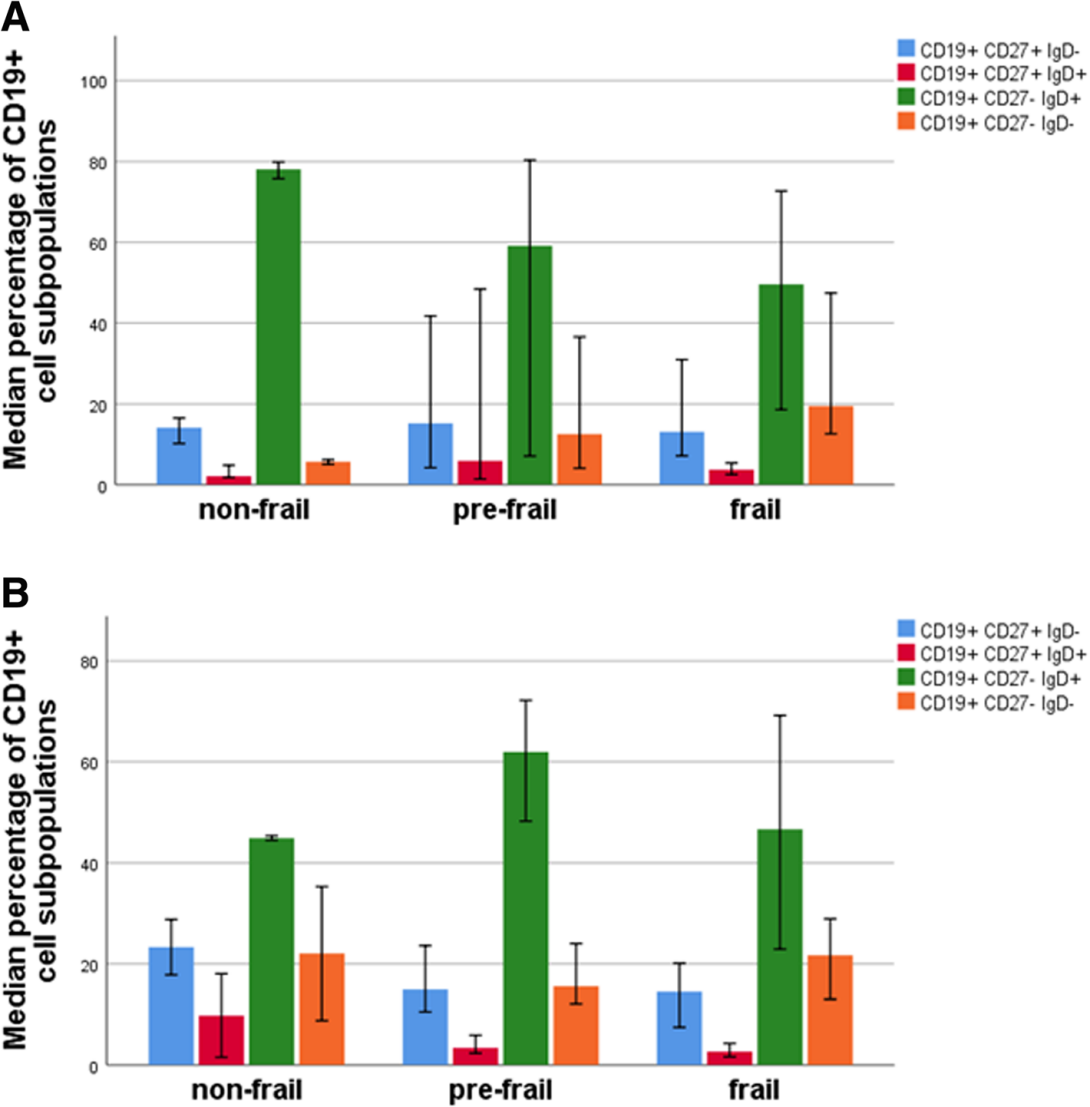
Distribution of CD19+ B cell subpopulations in nonagenarian individuals categorized with frailty index. **a** Nonagenarian males. **b** Nonagenarian females. Error bars represent 95% confidence interval

Gender did not have a significant effect on these indices (Barthel, mean 83 in females and 79 in males, frailty mean 2.1 in males and 2.5 in females). The original frailty index (min 0, max 5) was also modified to a 3 step index (0–1 = non-frail, 2–3 = pre-frail, 4–5 = frail). The number of nonagenarian male individuals in each frailty group were 3, 8 and 11 for the non-frail, pre-frail and frail, respectively, and the number of nonagenarian female individuals in each frailty group were 2, 20 and 17 for the non-frail, pre-frail and frail, respectively. The data shown in Fig. 4 shows that the percentage of the CD27- IgD- is ca. 3 times higher in the frail males than in the non-frails.

## Discussion

The findings reported here are in line with the previous data demonstrating that the CD27- IgD- B cell memory subset has a pro-inflammatory character. However, two novel aspects were observed. Firstly, its effect was so strong that there was a clear correlation with its increased proportion and the aging-associated decline of the physical capacity of the elderly individuals. Secondly, its effect was restricted to males.

As stated above, the CD19+ CD27- IgD- cells have a clear SASP phenotype, and are extensively characterized [5]. They are able to produce pro-inflammatory cytokines (tumor necrosis factor (TNF), IL-6, interleukin 8 (IL-8)) and contain typical inflammatory micro-RNAs. Obviously, they are already in a pre-activated stage. They also contribute to a functionally important parameter of immunosenescence, i.e. reducing the efficacy of vaccinations (at least in the case of influenzavirus) [14]. The data shown here that this cell type has a deleterious effect on the physiological reserves of the elderly individuals, as quantitated with the frailty index. It is unlikely, that a cell type with such a low frequency (less than 5% of the total PBMC number) would alone be responsible for such an effect. Thus, it is feasible that increase or activity of additional cell types with SASP phenotype would contribute to the observed effect. In fact, we addressed this question by analyzing the role of CD8+ CD28- T lymphocytes, i.e. a well-defined cell type with the SASP phenotype [15], in the same way as described here. However, the frequency of these cells did not correlate with any of the parameters used in this study (data not shown).

It has been shown that frailty is associated with several markers of inflammation, [16]. As frailty is a complex phenotype affecting many organs and tissues and probably also via various molecular pathways, it is obvious that it is not induced by a single molecule. In this study, we used IL-6 levels to quantitate the inflammation. IL-6 levels were associated much more weakly with the frailty index than the percentage of CD27- IgD- cells (data not shown), underlining the importance of yet uncharacterized factors in the pathogenesis of frailty.

The pro-inflammatory effect of the CD27- IgD- B cells was restricted to males. This is in line with our previous data [17]. In that study, we examined the correlation of IL-6 levels with transcriptomic and epigenetic changes in PBMCs of nonagenarians. In males, there were several transcripts correlating with IL-6 levels while in females there were none. These transcripts were involved in processes that were linked to inflammatory pathways. Thus, it seems likely, that the effect of the IL-6 component of inflammaging is gender-dependent. Obviously, this does not mean that all the effects of the CD27- IgD- cells would be restricted to males.

In this study, we used two indices, Barthel and frailty, to quantitate the aging-associated biological and functional deficiencies. Although the measured parameters or the questions asked in the calculation of these indices are different, they correlated significantly. This is not unexpected, as it is likely that decline of the biological capacity. i.e. frailty, probably leads to the functional deficiencies quantitated with the Barthel index. Although inflammaging associates with several disease states and pathogenic mechanisms (see above), it is probable that it is not functionally uniform entity, i.e. different forms of inflammaging have different targets. Individual analysis of the different assessment steps in the calculation of the frailty index might be informative in this respect. We observed that the CD27- IgD- proportion correlated very significantly with the muscle strength (the handgrip test), but only weakly with the cognitive status (MMSE test, data not shown). Thus, it is possible that the CD27- IgD- population associated inflammation affects preferentially the physical capacity (muscles, circulation).

The elderly individuals used in this study were nonagenarians, i.e. the biological aging process had progressed very far. It would be of interest to analyze the role of this CD27- IgD- cell type associated phenomena on the earlier stages of physical aging, e.g. at the age of 60–80 years, preferably in a follow-up setting. Unfortunately, such samples are not available in the Vitality 90+ cohort. A positive influence observed in these “younger” ones would maybe indicate that the effect of these CD27- IgD- cells is more general and would influence the pace of the decline of the physical functions. The reverse results, such that the findings would be restricted to nonagenarians, could be explained with differential inflammatory mediator profiles; the composition of the factors in the pool of the inflammatory mediators could be different between these age groups. However, in this study we used IL-6 as the marker of inflammation, i.e. the cytokine showing the male-restricted activity [17], while IL-1 levels, although increased in the nonagenarians, did not associate with the proportion of the CD27- IgD- cells (data not shown). Moreover, the central role of IL-6 in the present observations is further emphasized by its role as a biomarker, even an inducer of sarcopenia, (reviewed in [18]).

As for the male-specificity of the observed associations, it was quite surprising that the observed correlations between the proportion of CD27- IgD- cells and frailty and Barthel indexes were restricted to males. At present, the molecular/cellular background of this phenomenon is open and can only be speculated. Firstly, it is obvious that the number of the CD27- IgD- cells as such is not decisive, i.e. the proportion of these cells is even somewhat higher in females than in males (Fig. 2). Secondly, the production and effect of the proinflammatory cytokines could have a role. In this study, we used IL-6 as the indicator. Its serum levels were ca. two times higher in nonagenarian males than in females (Fig. 3), and correlated significantly with the proportion of the CD27- IgD- cells but only in males. Moreover, IL-6 levels demonstrated a weak correlation with the frailty index (*p* < 0.05 in males). We have previously shown that in male PBMCs IL-6 is a more potent activator than in female cells [17]. Thus, taken together, it is likely that both the IL-6 producer cell type, as well as the responsiveness to it, might be involved in the observed gender difference. Thirdly, it has been shown that the frailty index is gender-dependent to a certain extent, i.e. it is somewhat higher in females than in males of the same age [19] and the clinical consequences of its increase are different in males and females [20]. It is possible that these differences are due to the differences in the inflammatory response, or the causality could be reverse, i.e. the pathogenic changes associated with the advancing frailty would activate inflammation in a gender-specific manner.

As CD27- IgD- B cells seem to be very potent activators of inflammaging, even associating with adverse aging-associated effects, it is tempting to speculate that they could be a target for anti-aging therapies. In fact, Moura et al. [21] have recently shown that the increased number of CD27- IgD- cells in rheumatoid arthritis patients can be reduced back to normal levels with anti-TNF or anti-IL6 receptor antibodies.

## Conclusion

We observed that CD27- IgD- B cell subset demonstrates a strong pro-inflammatory effect in nonagenarian males, which significantly associates with the decline of the bodily functions. As CD27- IgD- B cells seem to contribute to inflammaging and its adverse effects, they might be an attractive target for anti-aging therapies.

## Data Availability

Data is available from the corresponding author on reasonable request.

## Abbreviations

FBS: Fetal bovine serum
IgD: Immunoglobulin D
IL-6: Interleukin 6
IL-8: Interleukin 8
MMSE: Mini-mental state examination
PBMC: Peripheral blood mononuclear cells
PBS: Phosphate-buffered saline
SASP: Senescence –associated secretory phenotype
TNF: Tumor necrosis factor
